# c-kit+ Cardiac Stem Cells Alleviate Post-Myocardial Infarction Left Ventricular Dysfunction Despite Poor Engraftment and Negligible Retention in the Recipient Heart

**DOI:** 10.1371/journal.pone.0096725

**Published:** 2014-05-07

**Authors:** Kyung U. Hong, Yiru Guo, Qian-Hong Li, Pengxiao Cao, Tareq Al-Maqtari, Bathri N. Vajravelu, Junjie Du, Michael J. Book, Xiaoping Zhu, Yibing Nong, Aruni Bhatnagar, Roberto Bolli

**Affiliations:** 1 Institute of Molecular Cardiology, Department of Medicine, University of Louisville, Louisville, Kentucky, United States of America; 2 Diabetes and Obesity Center, Department of Medicine, University of Louisville, Louisville, Kentucky, United States of America; University of Miami Miller School of Medicine, United States of America

## Abstract

Although transplantation of c-kit+ cardiac stem cells (CSCs) has been shown to alleviate left ventricular (LV) dysfunction induced by myocardial infarction (MI), the number of exogenous CSCs remaining in the recipient heart following transplantation and their mechanism of action remain unclear. We have previously developed a highly sensitive and accurate method to quantify the absolute number of male murine CSCs in female recipient organs after transplantation. In the present study, we used this method to monitor the number of donor CSCs in the recipient heart after intracoronary infusion. Female mice underwent a 60-min coronary occlusion followed by reperfusion; 2 days later, 100,000 c-kit+/lin- syngeneic male mouse CSCs were infused intracoronarily. Only 12.7% of the male CSCs present in the heart immediately (5 min) after infusion were still present in the heart at 24 h, and their number declined rapidly thereafter. By 35 days after infusion, only ∼1,000 male CSCs were found in the heart. Significant numbers of male CSCs were found in the lungs and kidneys, but only in the first 24 h. The number of CSCs in the lungs increased between 5 min and 24 h after infusion, indicating recirculation of CSCs initially retained in other organs. Despite the low retention and rapid disappearance of CSCs from the recipient heart, intracoronary delivery of CSCs significantly improved LV function at 35 days (Millar catheter). These results suggest that direct differentiation of CSCs alone cannot account for the beneficial effects of CSCs on LV function; therefore, paracrine effects must be the major mechanism. The demonstration that functional improvement is dissociated from survival of transplanted cells has major implications for our understanding of cell therapy. In addition, this new quantitative method of stem cell measurement will be useful in testing approaches of enhancing CSC engraftment and survival after transplantation.

## Introduction

A unique resident c-kit+ cardiac stem cell (CSC) population which is able to give rise to cardiovascular progenies has been previously identified and characterized in adult myocardium [1], [2], [3], [4], [5], [6], [7], [8]. Many studies have demonstrated the therapeutic potential of c-kit+ CSCs in alleviating ischemic cardiomyopathy. We and others have shown that transplantation of CSCs is effective in attenuating left ventricular (LV) dysfunction in animal models of both acute and chronic myocardial infarction (MI) [9], [10], [11], [12], [13]. Moreover, in a recent phase I clinical trial (SCIPIO), CSCs isolated from patients with ischemic cardiomyopathy have been found to significantly improve cardiac function, functional status, and quality of life when transplanted back into the patients via intracoronary infusion [14], [15], clearly demonstrating the utility of these cells for the treatment of ischemic heart failure.

However, there are still hurdles that need to be overcome to optimize CSC-based therapies. One of the major problems associated with cell therapy is the low rate of donor cell survival and engraftment in the recipient heart [16], [17], [18], [19], [20], [21], [22]. For example, in a mouse model of acute MI, Tang *et al*. reported that over 90% of the transplanted mesenchymal stem cells were lost during the first 24 h, and only 3.6% remained in the heart by 7 days post-transplantation [16]. In order to devise and test new approaches in CSC therapy, it is necessary to accurately monitor and compare the engraftment, retention, and fate of transplanted CSCs and correlate these variables with changes in cardiac function. However, most of the methods currently used to estimate or monitor the number of transplanted cells (which involve histological, *in vivo* imaging, or PCR-based techniques) permit only measurement of the relative changes in the transplanted cell number and do not provide information regarding the *absolute* number of cells present in a recipient organ of interest at a given time.

To overcome this problem, we have developed a novel quantitative, PCR-based method that allows measurement of the absolute number of donor CSCs with a high sensitivity and accuracy [23]. Using this method, we have shown that, after intramyocardial injection, CSC retention in the infarcted murine heart is very low [23]. CSC retention after intracoronary infusion (such as that used in SCIPIO [14]), however, is unknown. It is also unknown whether cell retention differs after intramyocardial and intracoronary administration. Finally, the relationship between number of cells persisting in the heart and cardiac function is unclear.

**Figure 1:**
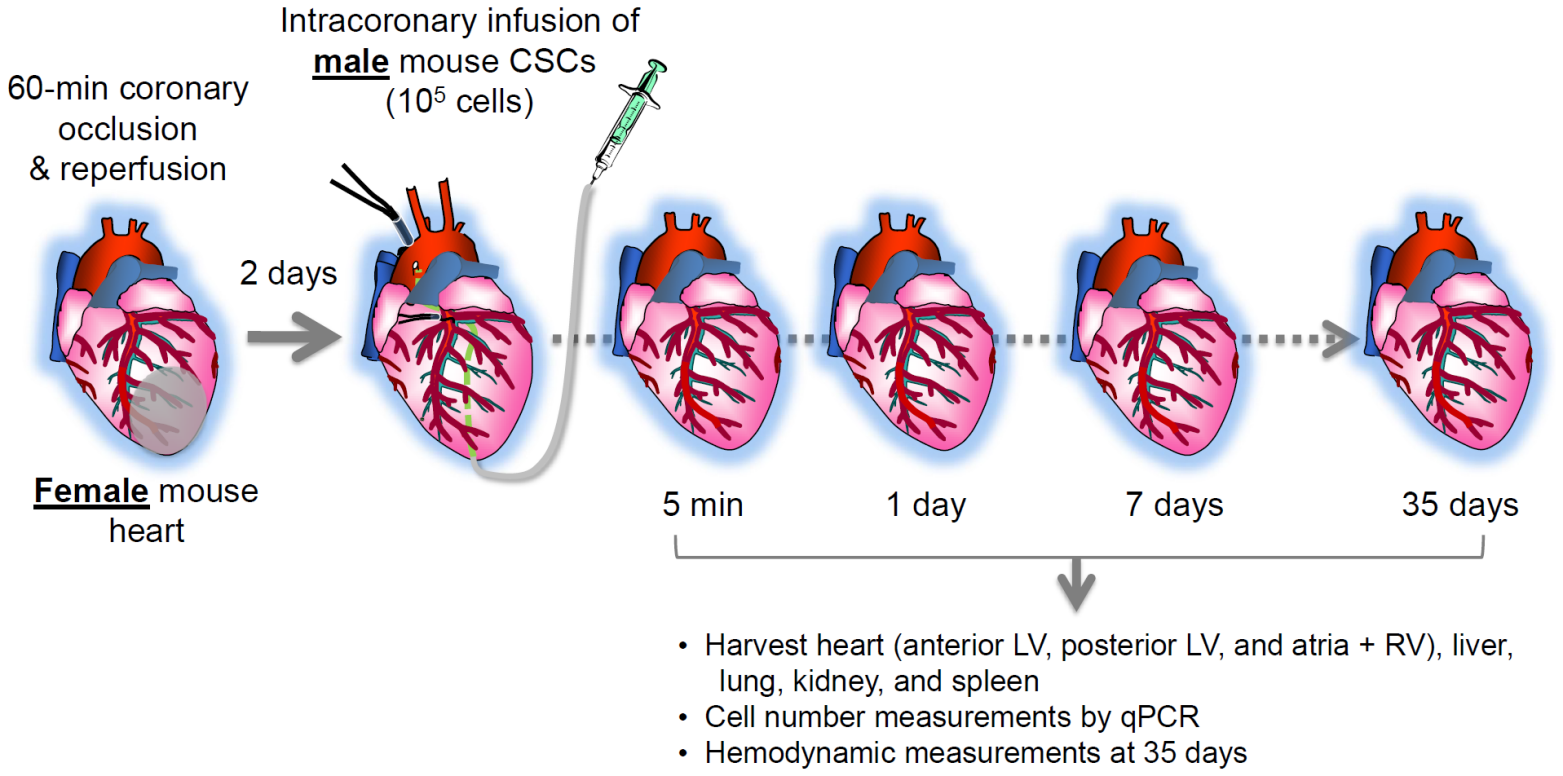
Experimental protocol. Left anterior coronary artery occlusion (60 min) followed by reperfusion was produced in female mice; two days later, mice received 10^5^ male murine CSCs (or PBS only) via intracoronary delivery. Heart, liver, lung, kidney, and spleen were collected at indicated time points. Each tissue was analyzed for the presence of donor CSCs by qPCR as described in Materials and Methods. In the 35-day group, hemodynamic measurements were performed prior to euthanasia to assess cardiac function.

The goal of the present study was to address these issues. To this end, we delivered male murine CSCs via the intracoronary route to female mice after MI and quantitatively assessed the distribution and retention of the transplanted cells in various recipient organs at serial time points (Fig. 1). Although intracoronary delivery of CSCs significantly improved LV function measured 35 days later, we found that CSCs engraftment was poor, and by 35 days cell retention in the recipient heart was negligible.

## Materials and Methods

### Ethics Statement

The present study was performed in accordance with the Guide for the Care and Use of Laboratory Animals (Department of Health and Human Services, Publication No. [NIH] 86–23) and with the guidelines of the Animal Care and Use Committee of the University of Louisville, School of Medicine. The protocol was approved by the Institutional Animal Care and Use Committee of the University of Louisville (Permit Number: 11064).

### Animals

This study was performed in C57BL6/J female mice (age 11–12 weeks), purchased from The Jackson Laboratory (Bar Harbor, ME). All mice were maintained in microisolator cages under specific pathogen-free conditions in a room with a temperature of 24°C, 55–65% relative humidity, and a 12-h light–dark cycle.

### Murine Model of Postinfarction LV Remodeling and Failure

The murine model of myocardial ischemia and reperfusion has been described in detail [24], [25]. Briefly, mice were anesthetized with sodium pentobarbital (60 mg/kg i.p.) and ventilated using carefully selected parameters. The chest was opened through a midline sternotomy, and a nontraumatic balloon occluder was implanted around the mid-left anterior descending coronary artery using an 8–0 nylon suture. To prevent hypotension, blood from a donor mouse was given at serial times during surgery. Rectal temperature was carefully monitored and maintained between 36.7 and 37.3°C throughout the experiment. In all groups, myocardial infarction (MI) was produced by a 60-min coronary occlusion followed by reperfusion. Mice were then allowed to recover for 5 min or 1, 7, or 35 days until euthanasia and tissue collection.

### Culture and Intracoronary Delivery of lin−/c-kit+ Mouse Cardiac Stem Cells

Isolation, characterization, and culture of lin−/c-kit+ mouse CSCs have been previously described [11]. Transplantation of mouse CSCs by intracoronary infusion was performed as described [11]. Briefly, at 48 h after reperfusion, mice were re-anesthetized, the chest reopened through a midline sternotomy, and the aortic root exposed by blunt dissection. A 30-gauge needle connected to a 100-µl syringe was advanced to the aortic root through the LV apex. The proximal aorta and pulmonary artery were cross-clamped with a snare for 10 s, during which time mice received an intracoronary infusion of CSCs (10^5^ cells in 80 µl of PBS) or vehicle (Fig. 1). After a clamp time of 10 s, aortic flow was restored.

### Isolation of Genomic DNA

After stem cell transplantation, mice were euthanized and the following organs were extracted at 5 min, 1 day, 7 days, or 35 days post-transplantation: heart, lung, liver, kidney, and spleen (Fig. 1). Upon extraction, the heart was separated into three different regions: anterior LV wall and septum (A LV) which included the infarcted and border zones, posterior LV wall (nonischemic region; P LV), and atria+right ventricle. Female human peripheral mononuclear cells (hPBMCs) were purchased from AllCells, LLC (Emeryville, CA). Aliquots of 10^5^ hPBMCs (in 50 µl PBS) were stored at −20°C until use. For isolation of genomic DNA, QIAamp DNA Mini Kit (Qiagen) was used according to the manufacturer’s instructions with the following modifications. In order to achieve complete digestion, the tissue samples were placed in screw-cap tubes containing an excess volume of Buffer ATL (tissue lysis buffer; 400 µl per 25 mg tissue) plus Proteinase K (40 µl per 25 mg tissue). The internal standard (10^5^ hPBMCs) was resuspended 50 µl of the above tissue digestion solution and added to each tube. The samples were incubated overnight at 56°C on a nutating or rotatory mixer. After the overnight incubation, the samples were treated with RNase A (Qiagen) to prevent RNA contamination prior to proceeding to the column purification of genomic DNA. Control DNA samples, including hPBMC, mouse CSC, and female mouse DNA, were prepared in a similar manner. The DNA concentrations of the samples were measured using a fluorometer (Qubit 1.0; Invitrogen) according to the instructions. For cardiac tissues, the entire tissue segment was used for DNA isolation. For extra-cardiac tissues, the whole organ was sonicated in a suitable volume of buffer, and the total homogenate was weighed. For each homogenate, 25–50 µl was taken out, weighed, mixed with 10^5^ hPBMCs and processed for genomic DNA isolation and qPCR analysis. The total number of male donor CSCs in the sample was then used to calculate the total number of CSCs in the entire organ based on the ratio of the weight of the processed tissue to the total organ weight.

### Real-time, Quantitative PCR

For each real-time PCR reaction, 100 ng of genomic DNA was amplified in a 20 µl reaction using SYBR Green PCR Master Mix and a StepOne Plus real-time PCR machine (Applied Biosystems). Each sample was run in triplicate and analyzed using three different sets of primers (*Rbmy*, *HLA-A,* and *Vim*). The primers used in the present study are the following: *Rbmy* fwd 5′-GACAAGAAGTGCTTCCACCA-3′; *Rbmy* rev, 5′-CAGCCCATCCTTAGGTGAAT-3′, *hHLA-A* fwd, 5′-GCTCAGTTCCAGTTGCTTG-3′; *hHLA-A* rev, 5′-GCAGTGAGCCAAGATTGCAC-3′; *Vim* fwd, 5′-GCAAGCCAGCCCACCTTCGA-3′; and *Vim* rev, 5′-ACCGGGTCACATAGGCGCCA-3′. qPCR for mouse vimentin gene (*Vim*) was run in order to ensure that a similar amount of total DNA is present in each sample. The PCR conditions were the following: 10 min at 95°C followed by 40 cycles of 2-step PCR (15 sec at 95°C and 1 min at 64°C for *hHLA* and *Vim* and 60°C for *Rbmy*). Melt curves were generated each time to validate the identity of the amplified products. The results were analyzed using StepOne Software v2.1 (Applied Biosystems). To generate hPBMC (human) DNA standards, 30, 100, 300, 1,000, 3,000, or 10,000 pg of hPBMC genomic DNA was added to 100 ng female mouse genomic DNA. To generate male mouse DNA standards, 10, 30, 100, 300, 1,000, 3,000, or 10,000 pg of male mouse CSC genomic DNA was added to 100 ng female mouse genomic DNA. Both sets of standards were analyzed by real-time PCR along with the samples, and the amount of target DNA (i.e., human or male mouse DNA) per 100 ng total DNA was plotted against the threshold cycle (C_T_) value to obtain the standard curves.

### Cell Number Calculations

The amount of target DNA (human or male mouse DNA) present in the PCR sample was calculated using the corresponding standard curve and was divided by the amount of genomic DNA present per cell (i.e., 7.02 pg for mouse and 6.71 pg for human) to calculate the number of target cells present in the 100 ng PCR sample. The total number of cells present in each tissue was calculated using the following equation: M = (m×H)/h in which M is the total number of male mouse cells in the tissue; m, the number of mouse cells present in the DNA sample; H, the total number of human cells added to the tissue sample (i.e., 10^5^); and h, the number of human cells present in the DNA sample [23].

### Hemodynamic Studies

In the 35-day vehicle and CSC groups, hemodynamic studies were performed just before euthanasia using the ARIA-1 system consisting of MPCU-200 P–V signal conditioning hardware, 1.0 French PVR-1035 microtip ultra-miniature pressure–volume (PV) catheters (Millar Instruments), data acquisition software, and PVAN data analysis software. Mice were anesthetized with isoflurane and rectal temperature kept between 36.7 and 37.3°C. A 1.0 French Millar catheter was inserted into the LV via the right carotid artery [26], [27]. Inferior vena cava occlusion was performed with external compression to produce variably loaded beats for determination of the end-systolic PV relation (ESPVR) and other derived constructs of LV performance. The raw pressure and volume data collected in text files by the MPCU-200 unit and Chart/Powerlab software were imported into the PVAN software, which applied a variety of algorithms to the P–V data to calculate up to 30 cardiovascular parameters [28], [29].

## Results

### Poor Retention and Rapid Disappearance of Transplanted CSCs Following Intracoronary Delivery into Infarcted Heart

**Figure 2:**
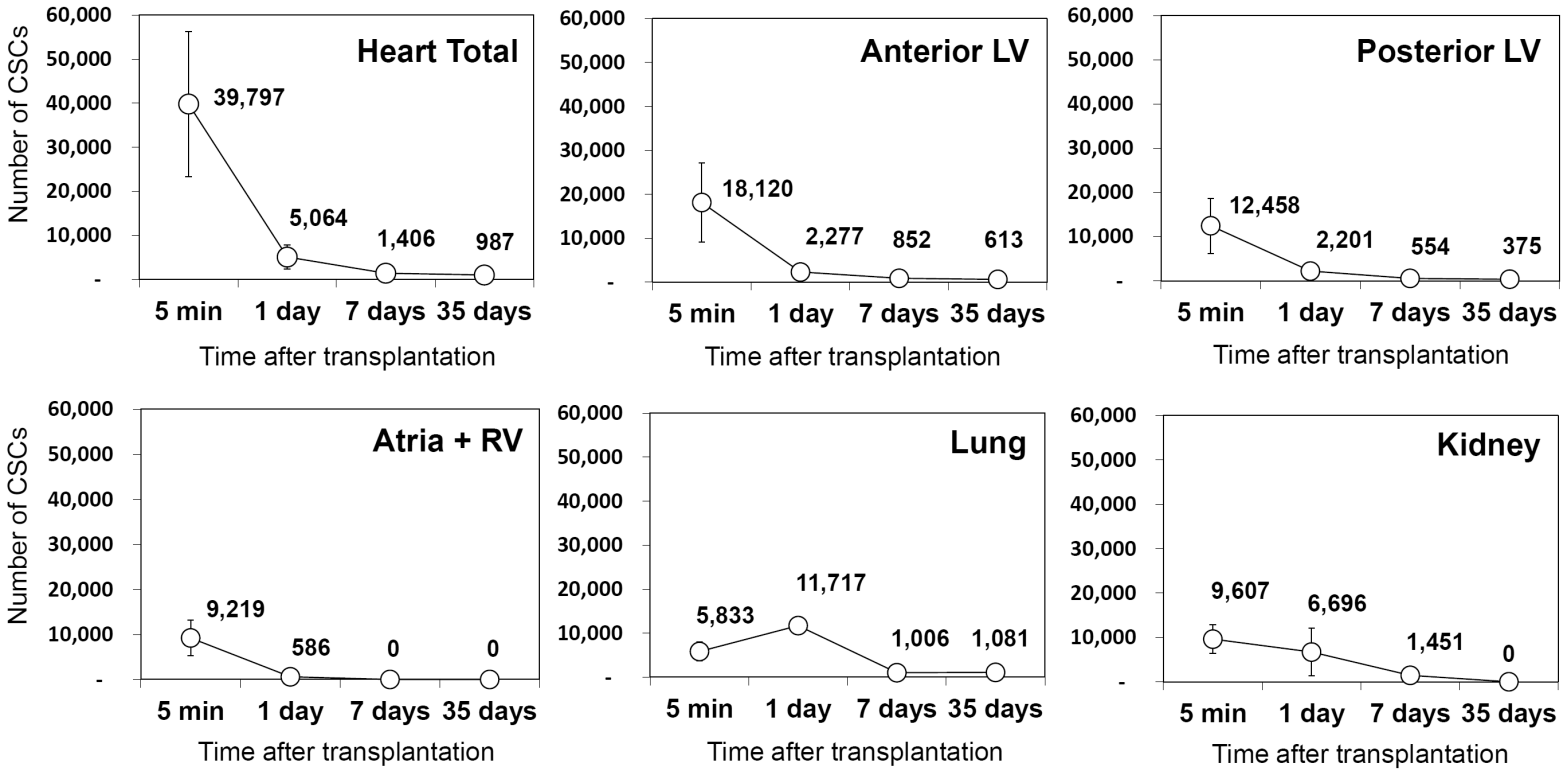
Distribution and retention of CSCs in various organs of the recipient mice. Values are means±SEM. CSCs could not be detected in liver and spleen samples at any time point examined. n = 4 for the first three time points and n = 7 for the 35 days group.

At 5 min after intracoronary infusion, <40% of the infused CSCs were found in heart (Fig. 2; Table 1), suggesting that most cells were washed off almost immediately by coronary blood flow. This early loss was expected. Importantly, however, >85% of the CSCs present in the heart at 5 min were lost in the ensuing 24 h, and only 3.5±2.0% of the CSCs present at 5 min could still be found in the heart at 7 days after transplantation (Fig. 2; Table 1). By 35 days, only 2.4±0.5% of the cells present at 5 min (i.e., 987±211 cells/heart, 1.0±0.2% of total cells injected) were detected in the heart (Fig. 2; Table 1).

**Table 1:** Number of transplanted CSCs found in the recipient tissues following intracoronary infusion of 10^5^ cells.

	Heart				Lung	Kidney
	Total	A LV	P LV	Atria+RV		
**5 min**	39,797±16,482	18,120±9,060	12,458±6,229	9,219±4,609	5,833±1,907	9,607±3,261
(n = 4)						
**1 day**	5,064±2,780	2,277±1,138	2,201±1,010	586±293	11,717±1,528	6,696±5,398
(n = 4)						
**7 days**	1,406±786	852±426	554±277	N.D.	1,006±1,006	1,451±1,451
(n = 4)						
**35 days**	987±211	613±232	375±142	N.D.	1,081±1,081	N.D.
(n = 7)						

Values are means ± SEM. A LV, anterior LV wall (including infarct and risk region); P LV, posterior LV wall; RV, right ventricle; N.D., non-detectable.

Throughout the observational period, the number of cells in the anterior LV wall (infarcted region) tended to be higher than that in the posterior LV wall, but the differences were not statistically significant. However, significantly fewer cells were initially found in the atria and right ventricle, and by 7 days post-infusion the cells were no longer detectable in these tissues. The only extra-cardiac organs that harbored significant numbers of CSCs were the lung and the kidney (Fig. 2, lower panels). The retention of cells in these two organs demonstrates that most of the infused CSCs passed through the coronary circulation and were distributed systemically. The number of CSCs in the lungs increased at 24 h vs. 5 min (Fig. 2; Table 1), indicating that many CSCs that were retained in the heart and other organs at 5 min after infusion became dislodged and entered the venous circulation in the ensuing hours. CSCs could not be detected in the liver and spleen at any time (data not shown).

### Intracoronary Infusion of CSCs Alleviates LV Dysfunction

**Figure 3:**
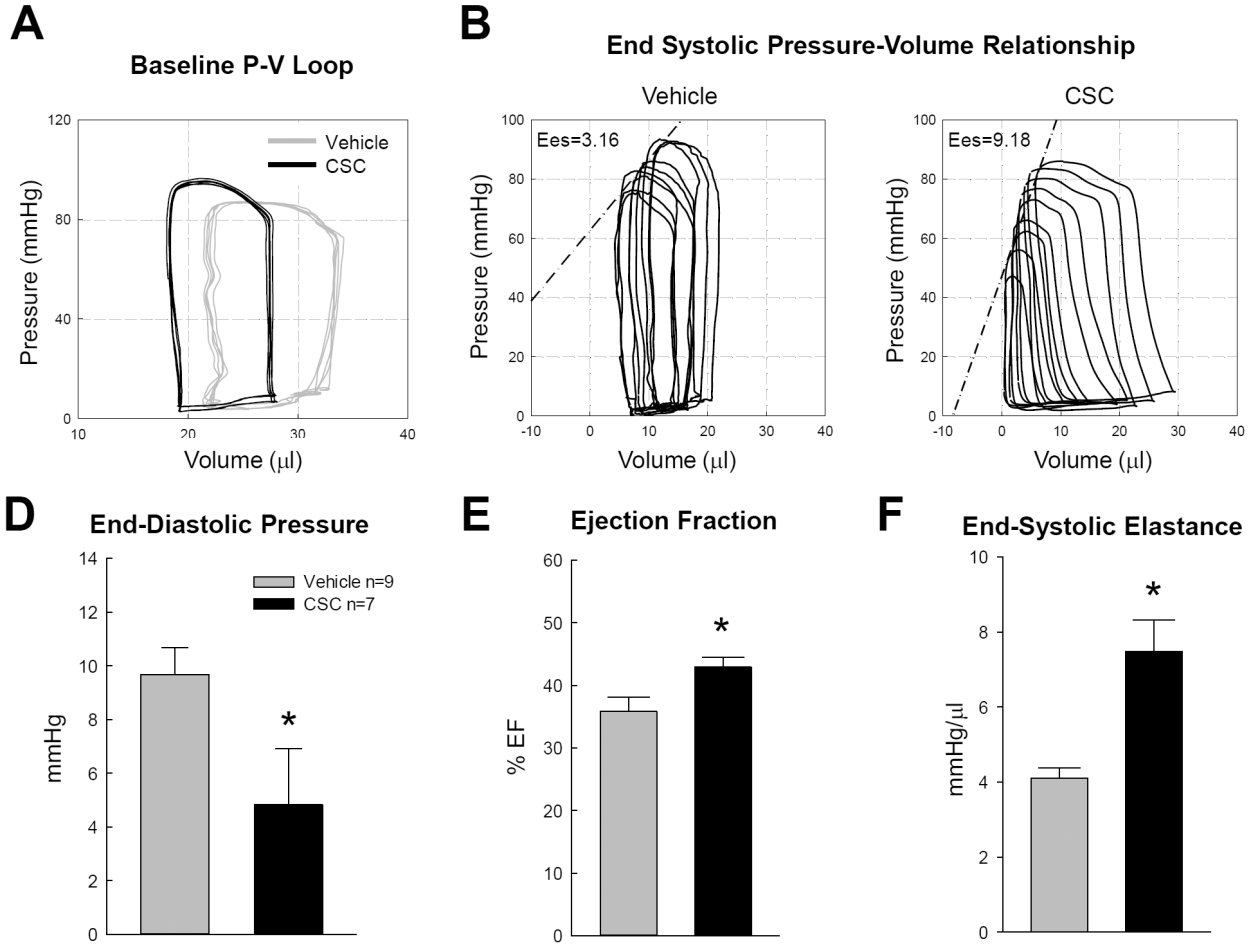
Intracoronary infusion of CSCs alleviates LV dysfunction induced by MI. At 35 days, hemodynamic measurements were performed using a Millar catheter in both vehicle- (n = 9) and CSC-treated mice (n = 7). A and B, Representative pressure-volume (P-V) loops from a vehicle-treated and a CSC-treated mouse recorded at baseline and during preload manipulation by a brief occlusion of the inferior vena cava. C, D, and E, Quantitative analysis of hemodynamic variables including LV end-diastolic pressure, ejection fraction and end-systolic elastance (Ees). Values are means±SEM. *, p<0.05 vs. vehicle group.

In order to assess the effect of intracoronary infusion of CSCs on cardiac function, vehicle- and CSC-injected mice were subjected to hemodynamic studies using a Millar catheter at 35 days after transplantation, prior to euthanasia and tissue harvest. Despite negligible retention of donor cells, the CSC-treated mice exhibited significantly lower LV end-diastolic pressures (4.81±2.22 mmHg vs. 9.62±1.06 mmHg in control mice) and greater LV ejection fraction (42.9±1.6% vs. 35.8±2.4%) and end-systolic elastance (a load-independent parameter of LV performance) (7.49±0.84 mmHg/µl vs. 4.11±0.27 mmHg/µl) (Fig. 3), indicating that the LV functional improvement that was dissociated from survival of transplanted cells.

## Discussion

In the present study, intracoronary infusion of 100,000 CSCs resulted in low initial retention (i.e., 39,797±16,482 cells at 5 min) in the heart with subsequent rapid disappearance of the cells. The vast majority (>85%) of the cells initially present in the heart (at 5 min after infusion) were lost during the ensuing 24 h, leaving only 5,064±2,780 cells at 1 day after infusion. The number of transplanted cells declined gradually thereafter, with only 987±211 cells (2.5±0.5% of those present at 5 min) left at 35 days after infusion. However, despite this poor retention and survival, LV function was significantly improved by CSC transplantation, as demonstrated not only by load-dependent but also by load-independent parameters of LV performance.

This is the first study to quantify the absolute number of donor CSCs that remain in the host after intracoronary infusion. The results are consistent with our previous studies of CSCs [9], [11], [23] (which used immunohistochemistry to assess cell retention) as well as with those obtained by others with other stem cells, all of which have shown low initial retention and almost complete disappearance after a few weeks [16], [17], [18], [19]. For instance, using a rat model of MI [9], we found a very low persistence of transplanted cells (or their progeny) at 35 days after transplantation, clearly insufficient to explain the functional improvement.

**Figure 4:**
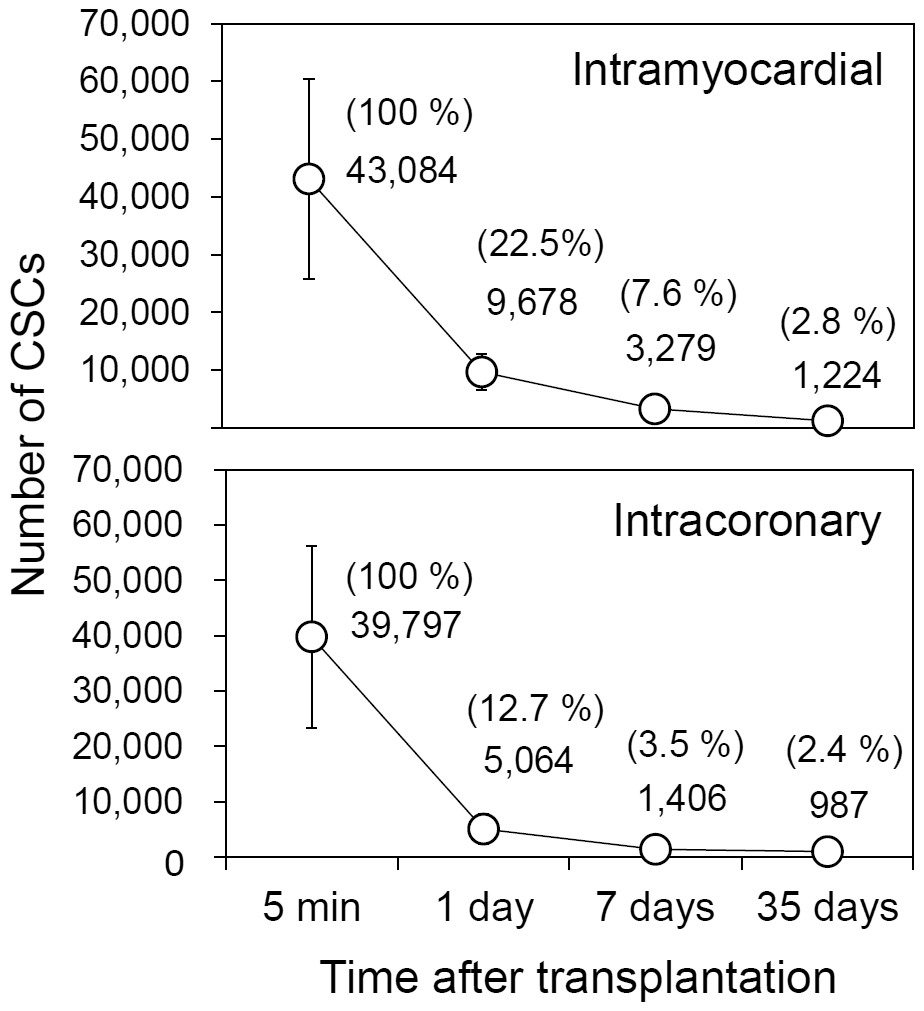
Comparison of CSC retention and engraftment after intramyocardial and intracoronary delivery. Female mice with acute MI were given 10^5^ c-kit+/lin- male murine CSCs by the intramyocardial [23] (upper panel) or intracoronary (present study; lower panel) route. The data shown in the top panel are reproduced from our previous paper [23]. The absolute numbers of CSCs detected in the entire heart at indicated time points are shown. The number of cells found at each time point, expressed as a percentage of the cells found at 5 min after delivery, is shown in the parenthesis. Data are means±SEM.

In a previous study [23], we employed the same assay and model system to monitor the retention and engraftment of CSCs (given at the same dose, 100,000 cells) following intramyocardial injection. We found that >55% of the injected cells were lost by 5 min, and that >75% of the CSCs present at 5 min were lost in the ensuing 24 h; only 7.6±2.1% of the CSCs present at 5 min could still be found at 7 days after injection and only 2.8±0.5% (i.e., 1,224±230 cells/heart) at 35 days (Fig. 4) [23]. Thus, even after direct intramyocardial injection, the number of CSCs that remain in the murine heart is minimal. The present study was performed to determine if a similar pattern applies to intracoronary infusion of CSCs. Our results show that the retention and engraftment of CSCs after intracoronary infusion parallel those seen after intramyocardial injection (Fig. 4). Comparison of intracoronary (present study) and intramyocardial (our previous study [23]) delivery of CSCs shows similar time-dependent changes in the number of cells remaining in the heart, although the initial retention appeared to be lower with intracoronary than with intramyocardial delivery (e.g., at 24 h, 12.7% of the cells present at 5 minutes were found after intracoronary infusion vs. 22.5% after intramyocardial injection; at 7 days, the corresponding percentages were 3.5% vs. 7.6%, respectively) (Fig. 4). Thus, regardless of the delivery route used, transplantation of CSCs in the infarcted murine heart is very inefficient and results in minimal cell retention.

Despite their negligible retention, CSCs significantly improved LV ejection fraction and end-systolic elastance compared with the vehicle control (Fig. 3). Because the number of CSCs remaining at 35 days was minuscule, differentiation of CSCs into new myocytes cannot explain the improvement in LV function, a conclusion that is also supported by our previous studies with intramyocardial injection of CSCs [11], [23]. In addition, we have repeatedly observed that only few transplanted cells express cardiomyocyte markers (e.g., α-sarcomeric actin), and that they do not acquire the phenotype of adult myocytes [9], [11], which makes it difficult to argue that cardiomyogenic differentiation of transplanted CSCs occurs in the recipient heart in a functionally meaningful manner. The clear dissociation between cell number and functional improvement observed here provided cogent evidence to support the concept that the major mechanism whereby the transplanted CSCs improve LV function must involve paracrine actions (i.e., production of signals [cytokines, miRs, microparticles, etc.] that modulate the function of adjacent cells).

One of the major problems inherent in previous qPCR-based methods is that they only allow measurement of the ratio of male donor to female recipient chimerism (which is often expressed as a percentage of male cells among female cells) [30], [31], [32], [33]. Thus, the absolute number of donor cells cannot be derived using such approaches unless the total number of cells present in the tissue/sample is known. There are two unique features that distinguish the current method from previous methods. The first is that the current method employs cells of another genotype (e.g., cells of human origin) which are added to each tissue sample as an internal standard prior to genomic DNA isolation. Since the ratio of the internal standard (i.e., human DNA) to the mouse DNA should remain constant regardless of the efficiency of DNA isolation, the amount of human DNA in each sample can be used to calculate the total, original amount of male donor DNA present in the entire tissue. The amount of DNA of a particular genotype can then be converted to the number of cells by dividing it by the amount of genomic DNA present in a cell (i.e., 6.71 pg for human and 7.02 pg for mouse diploid cells). The second feature of this method is that it targets a novel, male-specific, multiple-copy gene, *Rbmy* [23], [34], [35]. Although the sex-determining factor, *Sry* gene, has been traditionally used to identify cells of male origin, it is present in a single copy in both human and mouse Y chromosomes [36], [37], [38], and this limits the sensitivity of its detection. Thus, qPCR-based detection of a multiple-copy gene, *Rbmy*, significantly lowers the limit of detection of male DNA.

In summary, our data indicate that, after intracoronary infusion, murine CSCs exhibit low retention and engraftment, such that the absolute number of cells that remain in the heart after a few days is very small. Our data further demonstrate a clear dissociation between negligible persistence of CSCs in the heart and sustained functional improvement that persists long after most cells have disappeared. Thus, persistence of CSCs is not necessary for their beneficial effects to be manifest. These observations provide cogent evidence for a paracrine mechanism of action of CSCs, and have implications for future studies using the intracoronary route. In addition, the new quantitative method of stem cell measurement employed here will prove useful in testing approaches designed to enhance CSC engraftment and survival after transplantation and in understanding the mechanisms of CSC-based therapies.
